# Identification and Functional Analysis of Human CD56^neg^ NK Cells by Flow Cytometry

**DOI:** 10.1016/j.xpro.2020.100149

**Published:** 2020-10-29

**Authors:** Ane Orrantia, Iñigo Terrén, Joana Vitallé, Gabirel Astarloa-Pando, Olatz Zenarruzabeitia, Francisco Borrego

**Affiliations:** 1 Biocruces Bizkaia Health Research Institute, Immunopathology Group, 48903 Barakaldo, Spain; 2 Ikerbasque, Basque Foundation for Science, 48013 Bilbao, Spain

## Abstract

Although scarce in the peripheral blood of healthy people, CD56^neg^ NK cells are known to be expanded in some pathological conditions. However, studies on CD56^neg^ NK cells had revealed contradictions, probably due to the lack of a specific NK cell surface marker that helps to identify this subset. This protocol details the step-by-step procedure for the identification and functional analysis of CD56^neg^ NK cells, providing an improved gating strategy for the selection of this intriguing population.

For complete details on the use and execution of this protocol, please refer to Orrantia et al. (2020).

## Highlights

• Isolation of peripheral blood mononuclear cells from buffy coat
• Gating strategy for the identification of CD56^neg^ NK cells
• Analysis of the effector functions of CD56^neg^ NK cells

## Graphical Abstract

**Figure:**
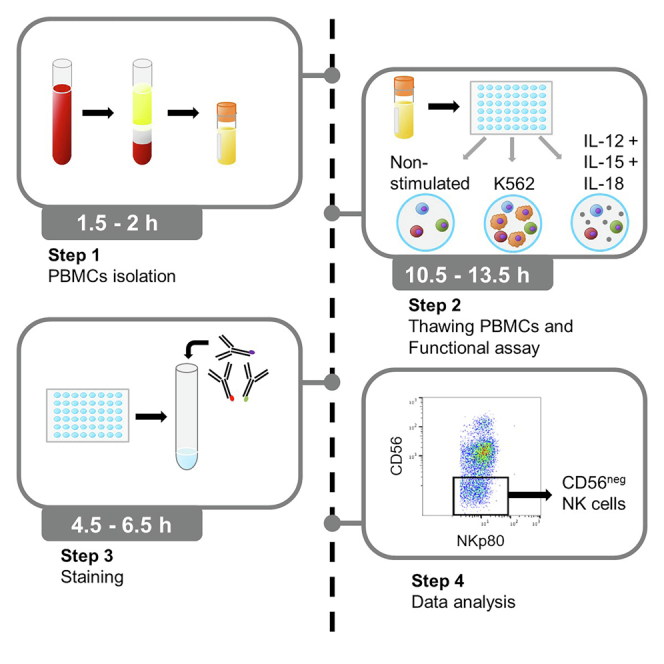

## Before You Begin

### Buffer and Media Preparation

**Timing: 0.5–4 h**

1. If needed, prepare the Phosphate Buffered Saline (PBS) and the PBS containing 2.5% of Bovine Serum Albumin (BSA) buffer. (See Materials and Equipment section).
2. If needed, prepare the 8% paraformaldehyde (PFA) solution in PBS. (See Materials and Equipment section).
3. If needed, prepare the culture media. (See Materials and Equipment section).

### Interleukins and DNase I Stock Preparation

**Timing: 1–3 h**

4. If needed, reconstitute each recombinant human interleukin (IL):
  a. IL-18 reconstitution:
    i. Add 250 μL of cold and filtered double distilled water (ddH_2_O) to 25 μg lyophilized IL-18 to obtain a stock concentration of 100 ng/μL.
    ii. For further dilution of the stock solution prepare sterile PBS + (0.1%) BSA, using sterile PBS and sterile BSA Fraction V (7.5% solution). (See Materials and Equipment section).
    iii. Dilute the IL-18 stock concentration adding sterile PBS + BSA (0.1%) to obtain a final working concentration of 25 ng/μL.
    iv. Store aliquots at −20°C.
  b. IL-12 reconstitution:
    i. Add 100 μL of cold and filtered ddH_2_O and 25 μL of sterile PBS + BSA (0.1%) to 25 μg lyophilized IL-12 to obtain a stock concentration of 200 ng/μL.
    ii. Dilute the stock concentration adding sterile PBS + BSA (0.1%) to obtain a final working concentration of 5 ng/μL.
    iii. Store aliquots at −20°C.
  c. IL-15 reconstitution:
    i. Add 100 μL of cold and filtered ddH2O and 150 μL of sterile PBS + BSA (0.1%) to 25 μg lyophilized IL-15 to obtain a stock concentration of 100 ng/μL.
    ii. Dilute the stock concentration adding sterile PBS + BSA (0.1%) to obtain a final working concentration of 5 ng/μL.
    iii. Store aliquots at −20°C.

***Note:*** Aliquoting the reconstituted ILs is recommended to avoid freezing/thawing cycles.

5. Reconstitute DNase I following manufacturer’s recommendation (https://www.sigmaaldrich.com/content/dam/sigma-aldrich/docs/Roche/Bulletin/1/11284932001bul.pdf). Briefly, reconstitute the lyophilized DNase I in sterile ddH_2_O to obtain a final stock concentration of 10 mg/mL (specific activity: 2,000 U/mg).

***Note:*** The working concentration of the DNase I is 10U up to 5 mL of media. We recommend aliquoting the stock solution to avoid freezing/thawing cycles. Store these aliquots at −20°C.

### Culture Cell Lines

**Timing: 1 week**

6. One week before starting the experiment, thaw cryopreserved K562 cells in a water bath at 37°C and put the cells in a conical 15 mL tube.
7. Add 3 mL of RPMI 1640 medium supplemented with L-Glutamine (Lonza) and centrifuge samples at 300–400 × *g* for 5 min at 20°C–22°C (room temperature (RT)). Discard supernatant.
8. Repeat step 7.
9. After washing, resuspend the pellet in cell line medium and plate the cells in a 25cm^3^ flask. Once the cells have expanded enough (after 2–3 days of culture), transfer them to a 75cm^3^ flask and let them expand for the experiment.

***Note:*** From a vial containing 10^7^ cryopreserved K562 cells, resuspend cells in a 15 mL tube with 10 mL of cell line medium (see Materials and Equipment section). Then, plate them at 1:6 dilution in a 25cm^3^ flask in a final volume of 10 mL (e.g., 1.6 mL cells in 8.4 mL of cell line medium)

***Note:*** We recommend to routinely test the K562 cell line for mycoplasma infection with Venor GeM Classic detection kit (Minerva BioLabs).

## Key Resources Table

**Table:** 

REAGENT or RESOURCE	SOURCE	IDENTIFIER
**Antibodies**		

BV421 Mouse monoclonal anti-CD56 (NCAM 16.2)	BD Biosciences	Cat# 562751; RRID: AB_2732054
BV510 Mouse monoclonal anti-CD3 (UCHT1)	BD Biosciences	Cat# 563109; RRID: AB_2732053
BV510 Mouse monoclonal anti-CD14 (MφP9)	BD Biosciences	Cat# 563079; RRID: AB_2737993
BV510 Mouse monoclonal anti-CD19 (SJ25C1)	BD Biosciences	Cat# 562947; RRID: AB_2737912
PerCP-Cy5.5 Mouse monoclonal anti-IFNγ (B27)	BD Biosciences	Cat# 560704; RRID: AB_1727532
APC Mouse monoclonal anti-TNF (MAb11)	BioLegend	Cat# 502912; RRID: AB_315264
PE monoclonal anti-CD107a (REA792)	Miltenyi Biotec	Cat# 130-111-699; RRID: AB_2654473
PE-Vio770 Mouse monoclonal anti-NKp80 (4A4.D10)	Miltenyi Biotec	Cat# 130-105-068; RRID: AB_2659831

**Biological Samples**		

Buffy coats from healthy adult donors	Basque Biobank for Research	n/a
Peripheral blood mononuclear cells (PBMCs) from multiple myeloma patients	Basque Biobank for Research	n/a
PBMCs from untreated HIV-1 infected subjects	HIV BioBank from the Spanish AIDS Research Network (RIS)	n/a
PBMCs from HIV-1 infected patients under combined antiretroviral therapy (cART)	HIV BioBank from the Spanish AIDS Research Network (RIS)	n/a

**Critical Commercial Assays**		

LIVE/DEAD™ Fixable Aqua Dead Cell Stain Kit for 405nm excitation	Invitrogen	Cat# L34957
Venor®GeM Classic	Minerva BioLabs	Cat# 11-1250

**Chemicals, Peptides, and Recombinant Proteins**		

Brilliant Stain Buffer	BD Biosciences	Cat# 563794
GolgiStop™ Protein Transport Inhibitor (monensin)	BD Biosciences	Cat# 554724
GolgiPlug™ Protein Transport Inhibitor (brefeldin A)	BD Biosciences	Cat# 555029
Perm/Wash™ Buffer	BD Biosciences	Cat# 554723
Paraformaldehyde (PFA)	Merck/ Sigma-Aldrich	Cat# P6148
Ficoll Paque Plus	GE Healthcare	Cat# GE17-1440-03
Fetal Bovine Serum (FBS)	GE Healthcare Hyclone	Cat# SV30160.03
Dimethylsulfoxide (DMSO)	Thermo Fisher Scientific	Cat# 20688
RPMI 1640 medium with L-Glutamine	Lonza	Cat# H3BE12-702F
DNase I	Roche (Merck)	Cat# 11284932001
RPMI 1640 medium containing GlutaMAX	Thermo Fisher Scientific	Cat# 72400054
Penicillin-Streptomycin	Thermo Fisher Scientific	Cat# 15140122
MEM Non-Essential Amino Acids Solution	Thermo Fisher Scientific	Cat# 11140035
Sodium Pyruvate (100 mM)	Thermo Fisher Scientific	Cat# 11360039
Plasmocin™ Treatment (25 mg/mL)	InvivoGen, IbianTechnologies	Cat# ant-mpt
Phosphate Buffered Saline (PBS) Tablets	Gibco, Thermo Fisher Scientific	Cat# 18912-014
Bovine Serum Albumin (BSA)	Millipore	Cat# A3733
Bovine Serum Albumin Fraction V in PBS (7.5% solution)	Thermo Fisher Scientific	Cat# 15260037
Trypan Blue Solution Cell Culture	Merck/ Sigma-Aldrich	Cat# T8154
Recombinant human Interleukin 15 (25 μg)	Miltenyi Biotec	Cat# 130-095-764
Recombinant human interleukin 12 (25 μg)	Miltenyi Biotec	Cat# 130-096-705
Recombinant human Interleukin 18 (25 μg)	MBL	Cat# B001-5

**Experimental Models: Cell Lines**		

Human K562 cell line	n/a	n/a

**Software and Algorithms**		

FlowJo™ Version 10.4.1	FlowJo LLC	https://www.flowjo.com/solutions/flowjo/downloads
GraphPad Prism v8.01	Prism-GraphPad	https://www.graphpad.com/scientific-software/prism/

**Other**		

MACSQuant Analyzer 10 flow cytometer	Miltenyi Biotec	n/a
Falcon® conical 50 mL centrifuge tubes	Falcon	Cat# 352070
70 μm cell strainers	Falcon	Cat# 352350
Corning® 15 mL PP Centrifuge Tubes	Corning	Cat# 430791
Falcon® 5 mL Round Bottom PP Test Tube	Falcon	Cat# 352002
Falcon® 48-well Clear Flat Bottom Not Treated Multiwell Cell Culture Plate	Falcon	Cat# 351178

## Materials and Equipment

***Alternatives:*** This protocol uses a MACSQuant Analyzer 10 flow cytometer for sample acquisition. Any other flow cytometer can be used as well. However, we recommend performing antibody titration to determine the optimal concentration of each antibody before starting the experiment.

**Table:** PBS

Reagent	Final Concentration	Amount
PBS Tablets	n/a	1 tablet
ddH_2_O	n/a	500 mL
**Total**	**n/a**	**500 mL**

Store at 4°C

**Table:** PBS + (2.5%) BSA

Reagent	Final Concentration	Amount
PBS	n/a	1 L
BSA	2.5% (w/v)	25 g
**Total**	**n/a**	**1 L**

Store at 4°C

**Table:** PBS + (0.1%) BSA

Reagent	Final Concentration	Amount
PBS	n/a	9.87 mL
Bovine Albumin Fraction V (7.5% solution)	0.1% (v/v)	133 μL
**Total**	**n/a**	**10 mL**

Store at 4°C

**Table:** 8% PFA

Reagent	Final Concentration	Amount
PBS	n/a	∼ 500 mL
PFA	8% (w/v)	40 g
**Total**	**n/a**	**500 mL**

Add 400 mL of PBS to a beaker and heat it until 55°C–60°C. Add 40 g of PFA and let it dissolve (it will take time). Allow the solution to cool down. Measure and adjust pH to 7 (add 1 M NaOH or HCl as needed). Filter the solution if needed. Add PBS to adjust the final volume to 500 mL. Measure and adjust pH to 7 again (add 1 M NaOH or HCl as needed). Store at 4°C.

**CRITICAL:** Note that PFA can be toxic as it is a potential carcinogen. To avoid inhalation of PFA vapors during solution preparation, it is recommended to perform this step in a certified chemical fume hood.

**Table:** R10 Medium

Reagent	Final Concentration	Amount
RPMI 1640 medium containing GlutaMAX	n/a	89 mL
FBS	10% (v/v)	10 mL
Penicillin-Streptomycin	1% (v/v)	1 mL
**Total**	**n/a**	**100 mL**

We filter sterilize (22 μm filter) the R10 medium after combining all the reagents. Store at 4°C. Do not store more than a month.

**Table:** NK Cell Medium

Reagent	Final Concentration	Amount
RPMI 1640 medium containing GlutaMAX	n/a	87 mL
FBS	10% (v/v)	10 mL
Penicillin-Streptomycin	1% (v/v)	1 mL
MEM Non-Essential Amino Acids Solution	1% (v/v)	1 mL
Sodium Pyruvate (100 mM)	1% (v/v)	1 mL
**Total**	**n/a**	**100 mL**

We filter sterilize (22 μm filter) the NK cell medium after combining all the reagents. Store at 4°C. Do not store more than a month.

**Table:** Cell Line Medium

Reagent	Final Concentration	Amount
RPMI 1640 medium containing GlutaMAX	n/a	217.5 mL
FBS	10% (v/v)	25 mL
Penicillin-Streptomycin	1% (v/v)	2.5 mL
MEM Non-Essential Amino Acids Solution	1% (v/v)	2.5 mL
Sodium Pyruvate (100 mM)	1% (v/v)	2.5 mL
Plasmocin Treatment	5 μg/mL	50 μL
**Total**	**n/a**	**250 mL**

We filter sterilize (22 μm filter) the cell line medium after combining all the reagents. Store at 4°C. Do not store more than a month.

***Note:*** Before use, we aliquot the plasmocin treatment in quantities of 50 μL. Store at −20°C.

***Note:*** The plasmocin treatment is added for preventing and eliminating mycoplasma contamination in cell cultures.

**Table:** Cell Cryopreservation Medium

Reagent	Final Concentration	Amount
FBS	n/a	4 mL
DMSO	20% (v/v)	1 mL
**Total**	**n/a**	**5 mL**

We recommend preparing this medium the day that it will be used and not to store it.

***Note:*** When adding the DMSO to the FBS, an exothermic reaction occurs. We recommend preparing the medium and wait some minutes (5–10 min) before add it to the cells. This medium contains 20% of DMSO, but it will be diluted with FBS to a final concentration of 10% DMSO when freezing the cells.

## Step-By-Step Method Details

### Peripheral Blood Mononuclear Cells (PBMCs) Isolation

**Timing: 1.5–2 h**

**Figure 1:**
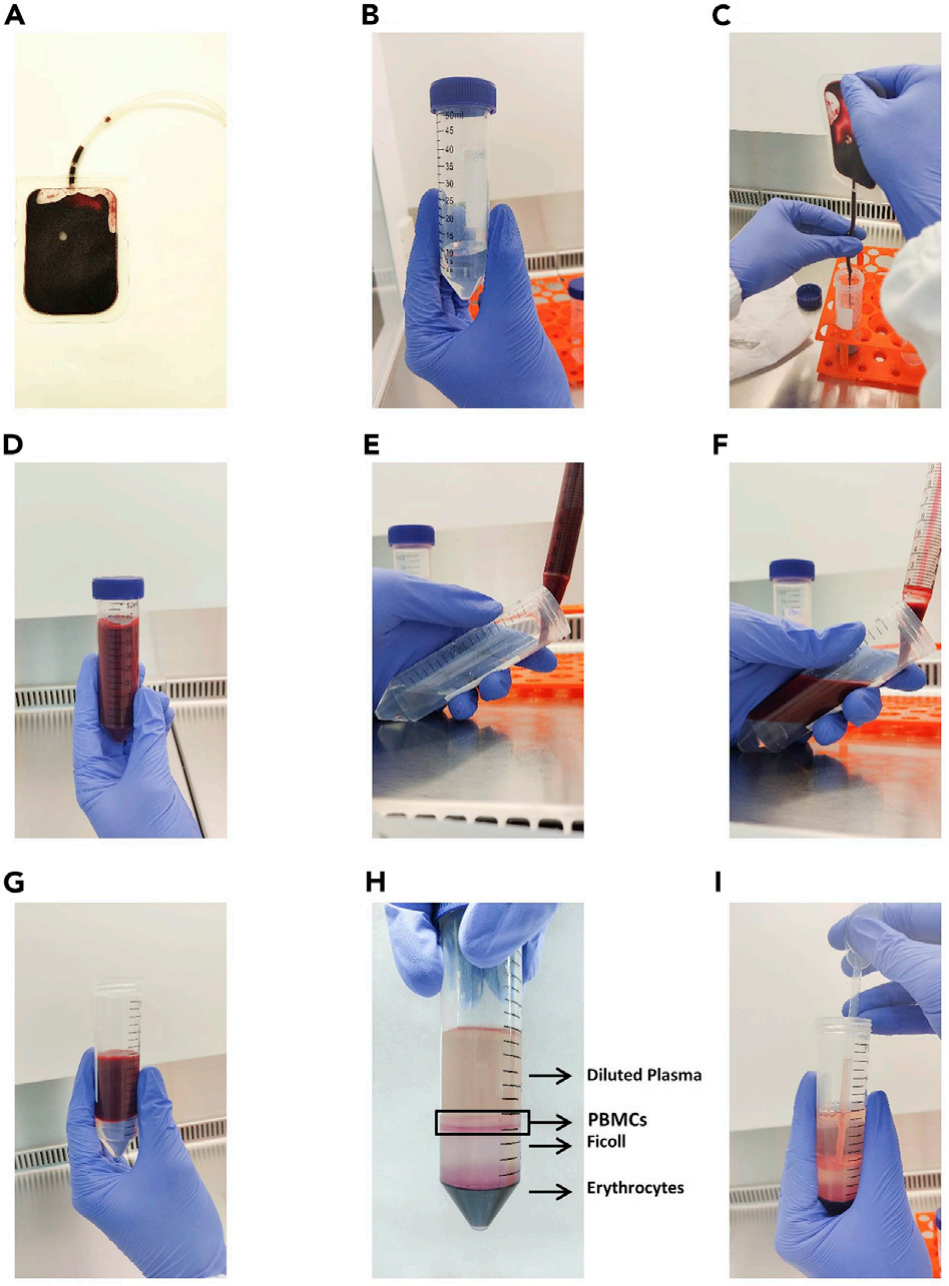
PBMC Isolation by Ficoll Density Gradient (A) Image of the buffy coat. (B) Add 10 mL of Ficoll in a conical 50 mL tube. (C) Add the buffy coat to another conical 50 mL tube. (D) Dilute the buffy coat 1:4 with PBS. (E–G) Add the diluted buffy coat on top of the Ficoll. (H) Image of Ficoll density gradient after centrifugation. (I) Collect the PBMC layer.

This step details how to isolate PBMCs from buffy coats (Figure 1A).

1. Add 10 mL of Ficoll Paque Plus to two conical 50 mL tubes (Figure 1B).
2. Dilute the buffy coat 1:4 by adding sterile PBS and mix gently. In our protocol, we add 30 mL of sterile PBS to 10 mL buffy coat (Figures 1C and 1D).

***Note:*** If starting from whole blood, prepare a 1:2 dilution in sterile PBS.

**CRITICAL:** During all the steps of PBMC isolation, use PBS without calcium and magnesium to avoid cell aggregation.

3. Carefully add 20 mL of the diluted buffy coat on top of the Ficoll in each conical tube (Figures 1E–1G).

**CRITICAL:** Avoid mixing the Ficoll and the diluted buffy coat. For that, tilt the conical tube with Ficoll and slowly add the diluted buffy coat or whole blood in the tube by sliding it through the tube wall.

4. Centrifuge tubes at 800 × *g* for 20–25 min at 20°C–22°C (RT).

**CRITICAL:** Use low brake and acceleration during the Ficoll density gradient centrifugation to avoid mixing of the layers.

5. Collect PBMC layer with a Pasteur pipette in a 50 mL conical tube (Figures 1H and 1I).
6. Add 30 mL of sterile PBS and centrifuge cells at 200 × *g* for 10 min at 20°C–22°C (RT).

***Note:*** This washing step is done at lower speed to remove platelets that could remain after collecting the PBMC layer.

7. After washing, discard the supernatant and resuspend the pellet in 30 mL of sterile PBS. Centrifuge samples at 300 × *g* for 10 min at 20°C–22°C (RT).
8. Repeat step 7.
9. Resuspend pellet in 20 mL sterile PBS and filter cells with 70 μm cell strainers.

***Note:*** The volume in which you should resuspend the pellet may vary depending on the concentration of your starting sample.

10. Count viable cells by Trypan Blue exclusion.
11. PBMCs cryopreservation:
  a. Prepare cell cryopreservation medium.
  b. We usually freeze 10^7^ cells per cryovial. Add the number of cells of interest in a conical 15 mL tube and centrifuge at 300 × *g* for 10 min at 20°C–22°C (RT).
  c. Discard the supernatant and resuspend the pellet in sterile FBS at a concentration of 500 μL/10^7^ cells.
  d. Place 500 μL of cells in each cryovial and add 500 μL of cell cryopreservation medium to obtain a final volume of 1 mL.
  e. Place all the vials in a freezing container and store it at −80°C for 48 h. Then, transfer the vials to an appropriate container and store them in liquid nitrogen.

***Note:*** The cryopreservation step must be done as quickly as possible. Try to reduce the time that cells are exposed to DMSO before they are transferred to the freezer as this influences cell viability.

***Note:*** The cryopreservation step can be omitted and fresh PBMCs can be used for phenotypic and functional assays. However, this protocol was optimized using cryopreserved samples. The cryopreservation of biological samples has some advantages such as, easier collection of the samples in locations far from where the study is performed, and possibility of analyzing samples in large batches, minimizing overall analytical variability.

### Thawing Cryopreserved PBMCs

**Timing: 2–3 h**

This step details how to thaw cryopreserved PBMCs before starting the phenotypical characterization and the functional assay.

12. Thaw the cryovial in a water bath at 37°C and place the cells into a conical 15 mL tube.
13. Add 3 mL of RPMI medium (previously warmed at 37°C) and centrifuge at 300–400 × *g* for 5 min at 20°C–22°C (RT).
14. Discard the supernatant and resuspended the pellet in 3 mL of R10 medium.
15. Add DNase I from the stock solution to obtain a working concentration of 10U DNase I for up to 5 mL cell suspension.
16. Place the cells in the incubator at 37°C and 5% CO_2_ for 1 h.
17. After the incubation, centrifuge the tubes at 300–400 × *g* for 5 min at 20°C–22°C (RT).
18. Discard the supernatant and resuspended the pellet in 2 mL of NK cell medium.
19. Filter cell suspension with a 70 μm cell strainer and place it into a new 15 mL tube.
20. Count viable cells by Trypan Blue exclusion.
21. If needed, add the appropriate volume of NK cell medium to obtain the desired concentration (e.g., 10^6^ cells/mL).

***Note:*** If phenotypic analysis are performed with the same sample, at this point divide the cells for the phenotypic and the functional analysis before continuing with the protocol.

### Functional Assay – Day 1

**Timing: 1–2 h**

This step details how to prepare the cells for the functional assay after the thawing process.

**Figure 2:**
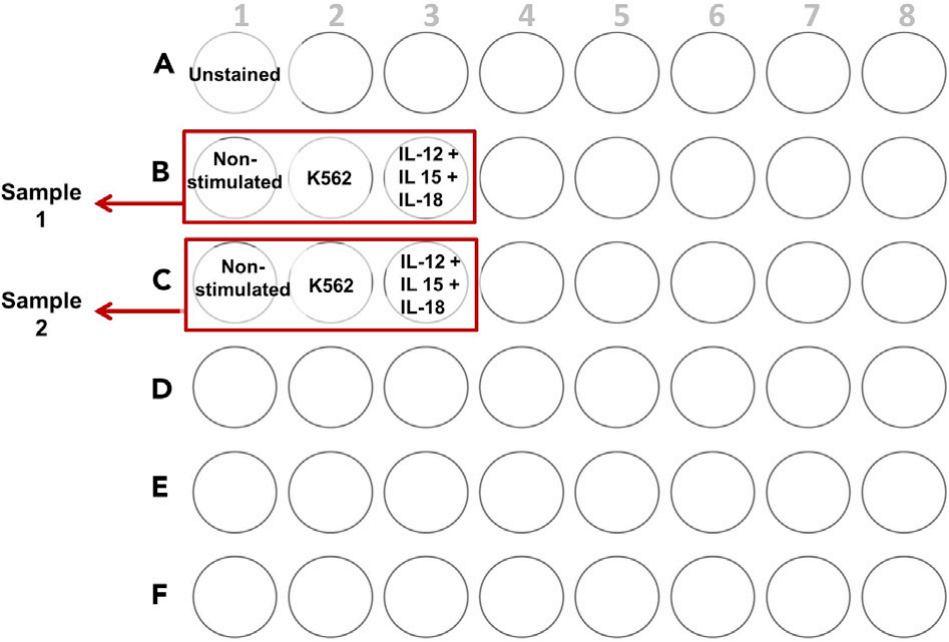
Schematic Representation of a Plate Layout for the Functional Assay

***Note:*** The functional assay consists of two different stimulation conditions: K562 cell line and IL-12+IL-15+IL-18 stimulation. In addition, a non-stimulated condition is needed as a control for each sample. Moreover, an unstained condition is needed for the whole experiment. The unstained condition serves as a control condition to check for cellular autofluorescence. Thus, this condition will be processed the same way as all the other conditions, except for the staining with the viability reactive dye and fluorochrome conjugated antibodies. (See Figure 2 for a plate set up example).

22. For each condition, plate 0.5 × 10^6^ of the thawed PBMCs in NK cell medium in a 48 wells plate. If needed, add the appropriate volume of NK cell medium to obtain a final volume of 1 mL/well.
23. Add 10 ng/mL of IL-15 to all the conditions.
24. Add 10 ng/mL of IL-12 and 50 ng/mL of IL-18 to the IL-12+IL-15+IL-18 stimulation condition.

***Note:*** We individually add each cytokine. However, preparing a master mix would also be fine.

25. Shake the plate very carefully by hand in a cross motion for 3 s to mix the wells. Then, incubate the cells in the incubator at 37°C and 5% CO_2_ until the next day.

***Note:*** In our protocol this incubation lasts for 20–21 h. However, shorter incubation time (17–18 h) may also work.

### Functional Assay – Day 2

**Timing: 7.5–8.5 h**

This step details how to perform the functional assay.

26. Prepare the K562 cell line for the NK cell stimulation:
  a. Collect the K562 cells from the 75cm^3^ flask used for their expansion and place cells in a conical 15 mL tube.
  b. Add RPMI 1640 medium with L-Glutamine (Lonza) for washing the cells.
  c. Centrifuge the cells at 300–400 × *g* for 5 min at 20°C–22°C (RT) and then discard the supernatant.
  d. Add again RPMI 1640 medium with L-Glutamine (Lonza).
  e. Count viable cells by Trypan Blue exclusion.
  f. Centrifuge the cells at 300–400 × *g* for 5 min at 20°C–22°C (RT) and then discard the supernatant.
  g. Resuspend the pellet in an appropriate volume of NK cell medium to get a final concentration of 2 × 10^6^ cell/mL.

***Optional:*** Take the plate with the PBMCs and centrifuge at 200 × *g* 1 min at 20°C–22°C (RT) with low brake and acceleration. This will fully ensure that PBMCs are settled in the bottom of the plate.

27. Take the plate and carefully remove 250 μL of medium from the K562 cell line condition avoiding PBMCs detaching from the bottom.
28. Add 0.5 × 10^6^ K562 cells (250 μL) to each K562 cell line condition well, to get a 1:1 Effector: Target (E:T) ratio.
29. Centrifuge the plate at 200 × *g* 1 min at 20°C–22°C (RT) with low brake and acceleration.
30. Add 20 μL/well of 1:10 diluted PE anti-CD107a monoclonal antibody (mAb) (REA792, Miltenyi Biotec, Cat# 130-111-699) to all the wells except to the unstained condition.

***Note:*** We prepare a final volume of 20 μL/well of anti-CD107a mAb by diluting it 1:10 with NK cell media. However, the manufacturer's recommended antibody dilution is 1:50 for up to 10^6^ cells or 100 μL. Therefore, we recommend doing a titration to determine the optimal antibody concentration.

31. Shake the plate very carefully by hand in a cross motion for 3 s to mix the wells. Then, incubate the plate in the incubator at 37°C and 5% CO_2_ for 1 h.
32. Individually add 0.66 μL/mL of monensin (GolgiStop) and 1 μL/mL of brefeldin A (GolgiPlug) to all the conditions. On the other hand, a master mix could be prepared before adding the two compounds to the wells.
33. Shake the plate very carefully by hand, in a cross motion, for 3 s to mix the wells. Then, incubate the plate in the incubator at 37°C and 5% CO_2_ for 5 more hours.

***Note:*** This protocol was optimized to continue with the staining steps (see Staining Day-1 section). However, it is also possible to stop the experiment at this point and continue with the staining the next day. For that, after the incubation period is over, take out the plate from the incubator and store it in the fridge (4°C), covered to protect from light, until the next day. Then, continue with step 34 of the protocol.

### Staining – Day 1

**Timing: 3–4 h**

This step details how to perform the extracellular staining and the fixation.

34. After the incubation, label cytometry tubes for each condition. Pipette up and down the cell suspension and transfer it from each well into tubes.

35. Viability staining:
  a. Add 2 mL of PBS to each tube and centrifuge at 300–400 × *g* for 5 min at 4°C.
  b. Discard the supernatant and resuspended pellet in 1 mL of PBS.
  c. If needed, reconstitute the LIVE/DEAD™ Fixable Aqua Dead Cell Stain Kit reactivity dye by adding 50 μL of DMSO to the vial. Mix well and visually confirm that all of the dye has dissolved.
    ***Note:*** We highly recommend performing live/dead staining when working with cryopreserved samples.
  d. Add 1 μL of the reactivity dye to all the tubes, except to the unstained condition, and vortex them. Leftover reconstituted viability dye can be frozen and used in future experiments following manufacturer's recommendations.
  e. Incubate for 30 min on ice. Protect from light.
  f. Add 2 mL of cold PBS + BSA (2.5%) to all the tubes and centrifuge at 300–400 × *g* for 5 min at 4°C. Discard the supernatant and resuspend the pellet in residual volume.

36. Prepare the antibody mix for the extracellular staining as follow (Table 1):

**Table 1:** Antibody Mix for Extracellular Staining

Fluorophore	Marker	Company	Cat#	Clone	Final Dilution	Volume
BV421	CD56	BD	562751	NCAM 16.2	1/33	3 μL/tube
BV510	CD3	BD	563109	UCHT1	1/33	3 μL/tube
BV510	CD14	BD	563079	MϕP9	1/33	3 μL/tube
BV510	CD19	BD	562947	SJ25C1	1/33	3 μL/tube
PE-Vio770	NKp80	Miltenyi Biotec	130-105-068	4A4.D10	1/20	5 μL/tube
	Brilliant Stain Buffer	BD	563794			50 μL/tube
	PBS + BSA (2.5%)					10 μL/tube
**Total**						77 μL/tube

***Note:*** The volume of each antibody has been optimized to acquire cells in the MACSQuant Analyzer 10 flow cytometer. We recommend performing antibody titration before starting the experiments.

***Note:*** We recommend preparing a bit more antibody mix volume than the indicated to ensure that the mix will be enough for all the tubes. For that, when doing the calculations, calculate for one additional tube or add a bit more of PBS + BSA (2.5%) before adding the mix to the tubes.

***Note:*** We recommend preparing the mix during viability dye incubation (step 35e) to save time.

**CRITICAL:** The BD Brillant Stain Buffer is a solution that is added when using two or more antibodies conjugated with BD Horizon Brillant fluorescence polymer dyes (e.g., BV421, BV510, etc.) to avoid fluorescent dye interaction, as these interactions may cause staining artifacts and affect data interpretation.

37. Extracellular staining:
  a. Add the corresponding volume of antibody mix (77 μL) to each tube, except to the unstained condition, and vortex them.
  b. Incubate for 30 min on ice. Protect from light.
  c. Add 2 mL of cold PBS + BSA (2.5%) to all the tubes and centrifuge at 300–400 × *g* for 5 min at 4°C. Discard the supernatant and resuspend pellet in residual volume.
38. Prepare 150 μL/tube of 4% PFA from the 8% PFA stock buffer by diluting it 1:2 with PBS.

***Note:*** We recommend preparing a bit more volume of 4% PFA buffer than what is needed.

39. Cell fixation:
  a. Add 140 μL/tube of 4% PFA and vortex.
  b. Incubate for 15 min on ice. Protect from light.
  c. Add 2 mL of cold PBS + BSA (2.5%) to all the tubes and centrifuge at 300–400 × *g* for 5 min at 4°C. Discard the supernatant and resuspend pellet in residual volume.
  d. Repeat step 39c.
40. Resuspend pellet in 1 mL of cold PBS + BSA (2.5%) and vortex.
41. Place the tubes in the fridge (4°C) protected from light until next day.

***Note:*** This protocol was optimized to stop the experiment at this point and continue the next day. However, it is also possible to continue performing the permeabilization and the intracellular staining steps. For that, omit steps 40 and 41 and continue as explained in step 44.

### Staining – Day 2

**Timing: 1.5–2.5 h**

This step details how to perform the permeabilization and the intracellular staining.

42. Take the tubes from the fridge and vortex them.
43. Centrifuge the tubes at 300–400 × *g* for 5 min at 4°C. Discard the supernatant and resuspend pellet in residual volume.
44. Prepare 3.5 mL/tube of 1× BD Perm/Wash™ Buffer by diluting the 10× BD Perm/Wash™ Buffer with ddH_2_O.
45. Cell permeabilization:
  a. Add 1 mL of 1× BD Perm/Wash™ Buffer to all the tubes and vortex.
  b. Incubate for 15 min at 20°C–22°C (RT). Protect from light.
  c. Centrifuge the tubes at 300–400 × *g* for 5 min at 4°C. Discard the supernatant and resuspend pellet in residual volume.
46. Prepare the antibody mix for the intracellular staining as follow (Table 2):

**Table 2:** Antibody Mix for Intracellular Staining

Fluorophore	Marker	Company	Cat#	Clone	Final Dilution	Volume
PerCP-Cy5.5	IFNγ	BD	Cat#560704	B27	1/20	5 μL/tube
APC	TNF	BioLegend	Cat#502912	MAb11	1/33	3 μL/tube
	1× BD Perm/Wash™ Buffer					50 μL/tube
**Total**						58 μL/tube

***Note:*** The volume of each antibody has been optimized to acquire cells in the MACSQuant Analyzer 10 flow cytometer. We recommend performing antibody titration before starting the experiments.

***Note:*** We recommend preparing a bit more antibody mix volume than the indicated to ensure that the mix will be enough for all the tubes. For that, when doing the calculations, calculate for one additional tube or add a bit more of 1× BD Perm/Wash™ Buffer before adding the mix to the tubes.

***Note:*** We recommend preparing the mix during cell permeabilization (step 45b) to save time.

47. Intracellular staining:
  a. Add the corresponding volume (58 μL) of antibody mix to each tube, except to the unstained condition, and vortex them.
  b. Incubate for 30 min on ice. Protect from light.
  c. Add 2 mL of 1× BD Perm/Wash™ Buffer to all the tubes and centrifuge at 300–400 × *g* for 5 min at 4°C. Discard the supernatant.
48. Resuspend the pellet with appropriate volume of PBS (200–400 μL) before acquiring the samples in the flow cytometer.

***Note:*** Before acquiring samples in the flow cytometer, we recommend performing fluorescence compensation to correct the emission spectra overlap of different fluorochromes following flow cytometer manufacturer’s recommendations. However, compensation can be also done after sample acquisition with different flow cytometry data analysis software (e.g., FlowLogic, FlowJo, etc.).

## Expected Outcomes

In this protocol, we have developed a gating strategy for the correct identification of human CD56^neg^ NK cells in peripheral blood and the subsequent functional analysis of this intriguing subpopulation.

**Figure 3:**
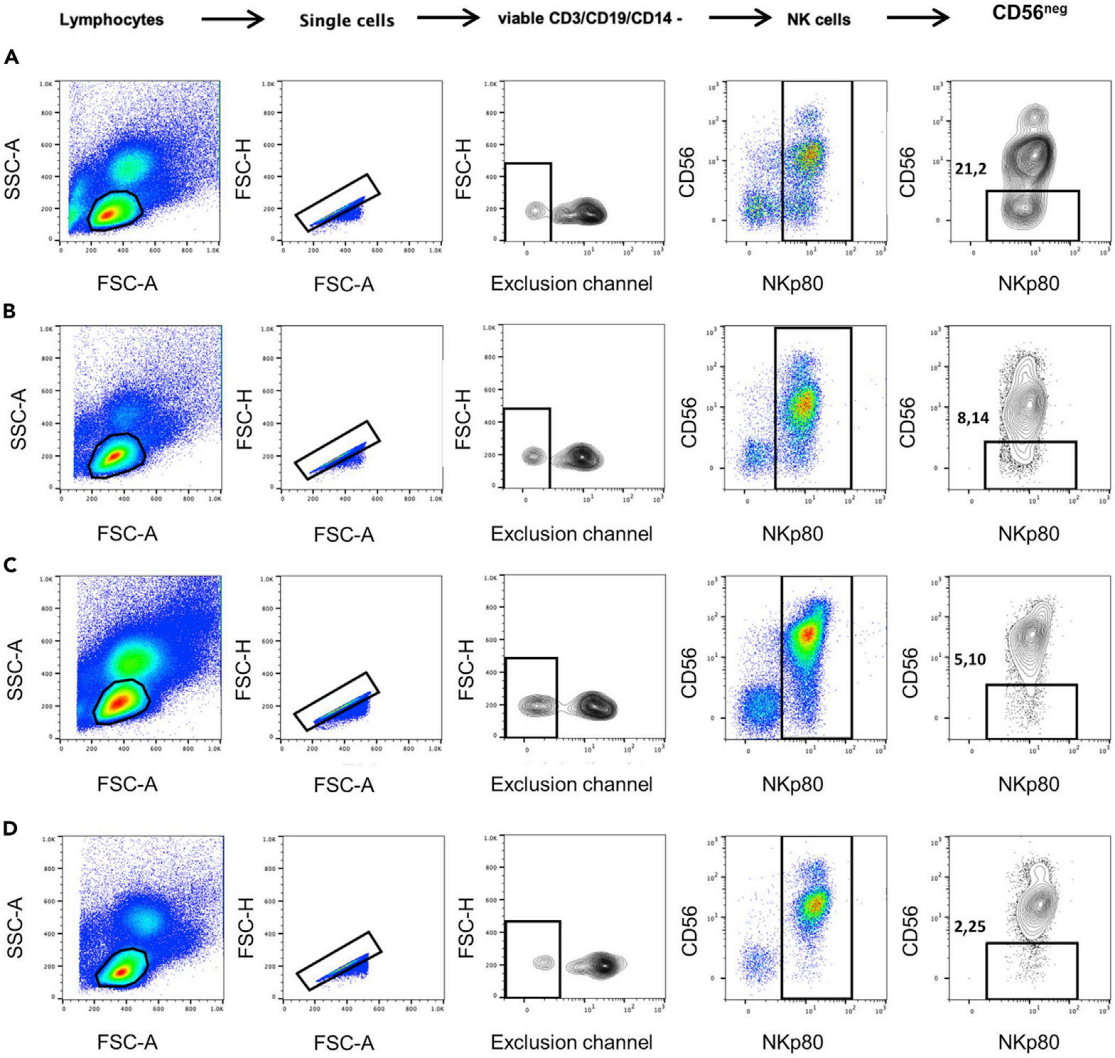
Gating Strategy for CD56^neg^ NK Cell Identification Pseudocolor and contour plot graphs representing the gating strategy utilized for the identification of CD56^neg^ NK cells. Data from a representative (A) untreated HIV-1 infected subject, (B) HIV-1 infected patient under cART, (C) multiple myeloma patient and (D) healthy individual are shown. Lymphocytes were electronically gated based on their forward and side scatter parameters and then single cells were selected. The population negative for the exclusion channel (viability, CD3, CD19, CD14) was selected for NK cell identification. The CD56^neg^ NK cells were identified as CD56^neg^NKp80+ cells.

First, we electronically gated lymphocytes based on their forward (FSC) and side (SSC) scatter parameters. Then single cells are selected based on FSC-area and FSC-height. An exclusion channel (viability dye and anti-CD3, anti-CD19 and anti-CD14 mAbs) was included in our gating strategy to specifically study non-T, non-B, non-monocytes viable cells. Thus, for NK cell identification, the negative population for the exclusion channel was selected. Lastly, CD56^neg^ NK cells were identified based on the expression of NKp80 and CD56 surface markers, as CD56^neg^NKp80+ cells (Figure 3). This population is known to be expanded in some pathological conditions: during human immunodeficiency virus (HIV)-1 infection in both untreated (Figure 3A) and in patients under combined antiretroviral therapy (cART) (Figure 3B) (Alter et al., 2005; Hu et al., 1995; Vitallé et al., 2019), and in multiple myeloma patients (Orrantia et al., 2020) (Figure 3C). CD56^neg^ NK cells can also be identified, although in lower frequencies, in healthy people (Figure 3D) (Campos et al., 2014; Müller-Durovic et al., 2019).

**Figure 4:**
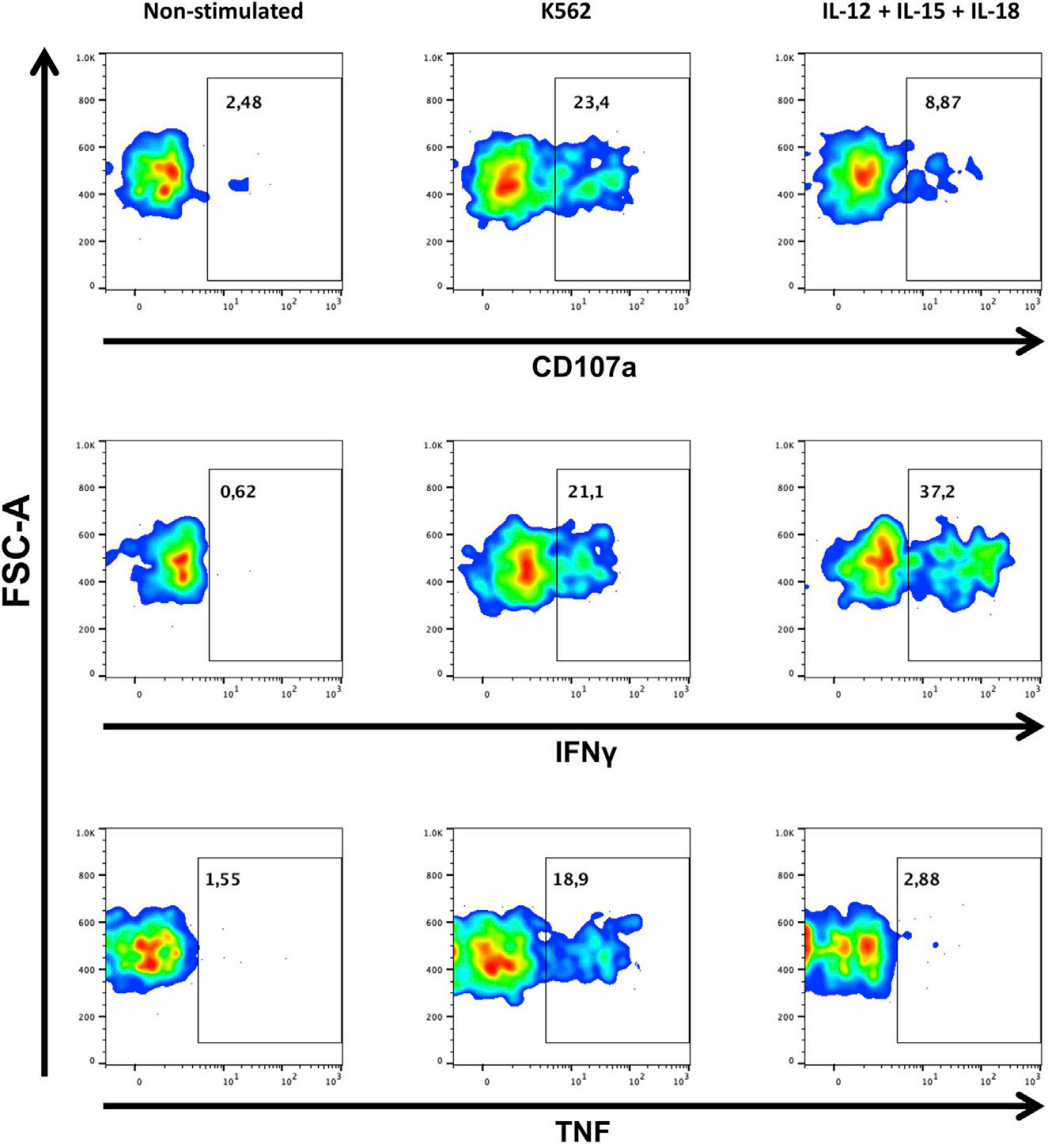
Effector Functions of CD56^neg^ NK Cells Pseudocolor graphs representing degranulation capability (CD107a) and production of IFNγ and TNF of CD56^neg^ NK cells in the IL-15 primed non-stimulated and K562 conditions and IL-12+IL-15+IL-18 condition of the functional assay. The percentage of cells positive for each marker is shown. Data from a representative cART HIV-1 infected subject is shown.

Once CD56^neg^ NK cells are correctly identified, the effector functions of this subset can be assessed. For that, we include anti-IFNγ and anti-TNF mAbs to determine cytokine production, and anti-CD107a mAb to measure degranulation capability of CD56^neg^ NK cells (Figure 4). This approach reveals that the effector functions of CD56^neg^ NK cells are not as diminished as previously described (Orrantia et al., 2020).

## Quantification and Statistical Analysis

To assess the effector functions of human CD56^neg^ NK cells we measure degranulation and production of TNF and IFNγ. We use FlowJo for the analysis of flow cytometry data.

1. Open the non-stimulated condition file and select CD56^neg^ NK cells as explained above (Figure 3).
2. Select the marker of interest (IFNγ, TNF, CD107a) and the FSC-A parameter and make a gate selecting the region where the population ends (Figure 4, left column). This will be the positive population for the marker of interest.

***Note:*** Alternatively, to determine the gate for the positive population, the fluorescence minus one (FMO) control can also be used. These controls contain all the fluorochrome conjugated mAbs, except for the one that is being measured in each case (IFNγ, TNF, or CD107a mAbs). FMO control is used to identify and gate cells in the context of data spread due to the multiple fluorochromes in a given panel. If you decide to use FMO controls you will need to prepare one control per marker that you want to determine and per condition (non-stimulated, K562 cell line and IL-12+IL-15+IL-18 conditions). Once you have the FMO controls, select CD56^neg^ NK cells as explained above (Figure 3) and determine the positive population for each marker in the corresponding FMO control.

3. Paste the positive gates for each marker in the K562 cell line and IL-12+IL-15+IL-18 conditions.
4. Calculate the percentage of positive cells for CD107a, IFNγ and TNF on each stimulation condition by subtracting the non-stimulated condition. We usually use Excel for this step.

## Limitations

This protocol uses NKp80 for the identification of CD56^neg^ NK cells. However, NKp80 is not a specific marker of NK cells because some CD8+ T cells also express it (Kuttruff et al., 2009), although these cells are gated out by using the exclusion channel. Within CD56^neg^NKp80+ subset, a small percentage of cells (around 20%) do not express Eomesodermin (Orrantia et al., 2020), a transcription factor needed for the development and function of NK cells (Vivier et al., 2018). On the other hand, although NKp80 is not downregulated on our stimulation protocol (Orrantia et al., 2020), Klimosch et al. have described that NK cells stimulated with PMA or IL-2+IL-12+IL-18 downregulated the expression of this receptor after 24 h stimulation (Klimosch et al., 2013). This suggests that NKp80 is not the perfect surface marker to identify the intriguing CD56^neg^ NK cell subset and further studies are needed to identify a specific NK cell surface marker. Nevertheless, the gating strategy we proposed in this protocol is the best currently available for the identification and subsequent analysis of the effector functions of CD56^neg^ NK cells, especially when it concern to frozen samples.

## Troubleshooting

### Problem 1

Flow cytometer laser and filters configuration do not allow for the detection of the proposed fluorochromes (steps 36 and 46).

### Potential Solution

To overcome this problem, try to find the same antibody clones conjugated with fluorochromes that your flow cytometer can detect. Moreover, we recommend choosing a fluorochrome with a high stain index for CD56 marker since it is important to be able to differentiate between CD56^dim^ and CD56^neg^ NK cells.

### Problem 2

Inadequate cell number for functional assay (step 22).

### Potential Solution

This protocol was optimized for using 1.5 × 10^6^ PBMCs per sample for functional assay (0.5 × 10^6^ PBMCs per condition). However, if you have less cells, we propose three different potential solutions:

• Using 96 U-bottom wells plates instead of 48 wells plates is also possible. In this event, 0.2 × 10^6^ PBMCs are plated per condition in a final volume of 200 μL. Be aware that you will need to recalculate the volume of the three ILs and GolgiStop and GolgiPlug that you must add to each well. In addition, we recommend doing the titration of the anti-CD107a mAb, although adding 2 μL/well may work. Maintain the E:T ratio of the K562 cell line stimulation condition in 1:1.
• You can also decide to not include one of the stimulation conditions, as for example the IL-12+IL-15+IL-18 condition. This condition is principally used for the measurement of IFNγ production but you should also be able to measure production of this cytokine (although at lower levels) with the K562 cell line condition.
• Considering that the unstained condition is only used as a control, you can always plate fewer cells. Using 0.25–0.3 × 10^6^ PBMCs for the unstained condition may also work.

## Resource Availability

### Lead Contact

Further information and request for resources and reagents should be directed to and will be fulfilled by the Lead Contact, Francisco Borrego (francisco.borregorabasco@osakidetza.eus).

### Materials Availability

This study did not generate new unique reagents.

### Data and Code Availability

This study did not generate datasets and codes.
